# Corn planting and harvest scheduling under storage capacity and growing degree units uncertainty

**DOI:** 10.1038/s41598-022-25797-9

**Published:** 2022-12-28

**Authors:** Zahra Khalilzadeh, Lizhi Wang

**Affiliations:** Department of Industrial and Manufacturing Systems Engineering, Iowa State University, Ames, IA 50011 USA

**Keywords:** Computational biology and bioinformatics, Plant sciences

## Abstract

Planting and harvest scheduling is a crucial part of crop production due to its significant impact on other factors such as balancing the capacities for harvest, yield potential, sales price, storage, and transportation. Corn planting and harvest scheduling is challenging because corn hybrids have different planting windows, and, subsequently, inaccurate planting and harvest scheduling can result in inconsistent and unpredictable weekly harvest quantities and logistical and productivity issues. In the 2021 Syngenta Crop Challenge, participants were given several large datasets including recorded historical daily growing degree units (GDU) of two sites and provided with planting windows, required GDUs, and harvest quantities of corn hybrids planted in these two sites, and were asked to schedule planting and harvesting dates of corn hybrids under two storage capacity cases so that facilities are not over capacity in harvesting weeks and have consistent weekly harvest quantities. The research problem includes determining the planting and harvest scheduling of corn hybrids under two storage capacity cases: (1) given the maximum storage capacity, and (2) without maximum storage capacity to determine the lowest storage capacity for each site. To help improve corn planting and harvest scheduling, we propose two mixed-integer linear programming (MILP) models and a heuristic algorithm to solve this problem for both storage capacity cases. Daily GDUs are required for planting and harvest scheduling, but they are unknown at the beginning of the growing season. As such, we use recurrent neural networks to predict the weekly GDUs of 70 weeks and consider this as the predicted GDU scenario to solve this problem. In addition, we solve this problem considering all given 10 historical GDU scenarios from 2010 to 2019 together for both storage capacity cases to include historical GDUs directly to our model rather than using predicted GDUs. Our extensive computational experiments and results demonstrate the effectiveness of our proposed methods, which can provide optimal planting and harvest scheduling considering deterministic GDU scenario and uncertainties in historical GDU scenarios for both storage capacity cases to provide consistent weekly harvest quantities that are below the maximum capacity.

## Introduction

The advent of new farming technologies such as commercial hybrids in the 1930s preceded a widespread and rapid replacement of the once predominant open-pollinated seed varieties planted by farmers^1^. The widespread use of commercial hybrids is seen in many crops, including corn, sorghum, sugar beet, and sunflower^2^. Of these crops, corn is widely known as one of the world’s most produced and important crops.

Scheduling planting and harvesting dates of corn hybrids is an important part of corn crop production. Accurate scheduling not only allows corn ears sufficient time to reach maturity but also keeps a consistent harvest amount under the storage capacity. Poor scheduling may result in inconsistent harvest quantities which can cause growers to experience logistical challenges. Moreover, it can result in having harvest quantities above the maximum capacity which might lead to either dump harvested crops or leave crops unharvested resulting in a financial loss. Growing Degree Days (GDD), also known as Growing Degree Units (GDU) or heat units, are a measure of accumulation of heat or temperature units used to estimate the plant growth stage. GDUs are calculated based on air temperature by subtracting the base temperature from the average of the daily maximum and minimum air temperatures^3^:

1 $$\begin{aligned} \text {GDU} = \text {(Daily maximum air temperature} + \text {Daily minimum air temperature})/2 - \text {Base air temperature.} \end{aligned}$$

Base temperature is the temperature below which the crop does not grow, and it is different for different species and varieties. In the case of corn, ${50}\,^{\circ }$F (${10}\,^{\circ }$C) is often used as the base temperature. If the daily maximum temperature is above ${86}\,^{\circ }$F (${30}\,^{\circ }$C), then the daily maximum temperature is set at ${86}\,^{\circ }$F (${30}\,^{\circ }$C) as above that temperature the growth rate of corn is not significant. Likewise, when the daily minimum temperature is less than ${50}\,^{\circ }$F (${10}\,^{\circ }$C), then this value is set at ${50}\,^{\circ }$F (${10}\,^{\circ }$C)^4^.

Many factors affect the planting date of crops, such as weather, soil temperature, and planting resources. Traditionally, farmers and growers schedule the crops’ planting dates to have a continuous harvest at the end of the growing season. Such a planting schedule has multiple benefits: (1) farmers do not have to harvest all crops at one time, and (2) farmers have some control of the harvest quantity in response to market fluctuation of crop prices to maximize their profit^5^.

More recently, deep learning techniques have been utilized in many agricultural big data applications including crop yield prediction, classification of crop tolerance to heat and drought, and image-based crop yield estimation^6–13^. There are very few studies in the literature that use optimization models to obtain optimal planting or harvest schedule. In crop planning, Cid-Garcia et al. and Sarker et al. proposed a linear programming model to help farmers decide how to dedicate different parts of their land to different crops at different points of time to maximize their profit^14,15^. To the best of our knowledge our paper is the first study to propose mixed integer linear programming (MILP) models which result in optimal planting and harvesting dates for different storage capacity cases considering different GDU scenarios.

In the 2021 Syngenta Crop Challenge^16^, Syngenta provided real-world data and asked participants to use the data to determine optimal planting and harvest schedules of corn hybrids for two storage capacity cases. The goal is to harvest corn hybrids in a minimum number of weeks and have consistent weekly harvest quantities under the storage capacity. In this paper, we propose our approach to the 2021 Syngenta Crop Challenge. We designed two MILP models and a heuristic algorithm for two storage capacity cases, which provide optimal planting and harvest schedules while ensuring consistent weekly harvest quantities under the storage capacity. Our proposed optimization models consider both the predicted GDU scenario and all 10 historical GDU scenarios to provide optimal planting and harvest schedules. We solved the MILP models for the predicted GDU scenario using the Gurobi MILP solver and implemented the heuristic algorithm in Python for multiple GDU scenarios.

The rest of this paper is structured as follows. “Data” section describes the data used in this research. “Methods” section provides a detailed description of our proposed MILP models and heuristic algorithm. “Results” section presents the results of our proposed models for two storage capacity cases, and two GDU scenarios including predicted GDU scenario, and all 10 GDU scenarios together. In “Evaluation of the proposed heuristic algorithm” section, we evaluate the performance of the proposed heuristic algorithm (1) by solving the corn scheduling problem considering multiple GDU scenarios using the proposed heuristic algorithm (1) and more generalized MILP model (19)–(25) for a small subset of populations from site 1 for the storage capacity case 1 and comparing their results. Finally, the study is concluded by summarizing the key results, findings, and directions of future work in “Discussion and conclusion” section.

## Data

**Figure 1:**
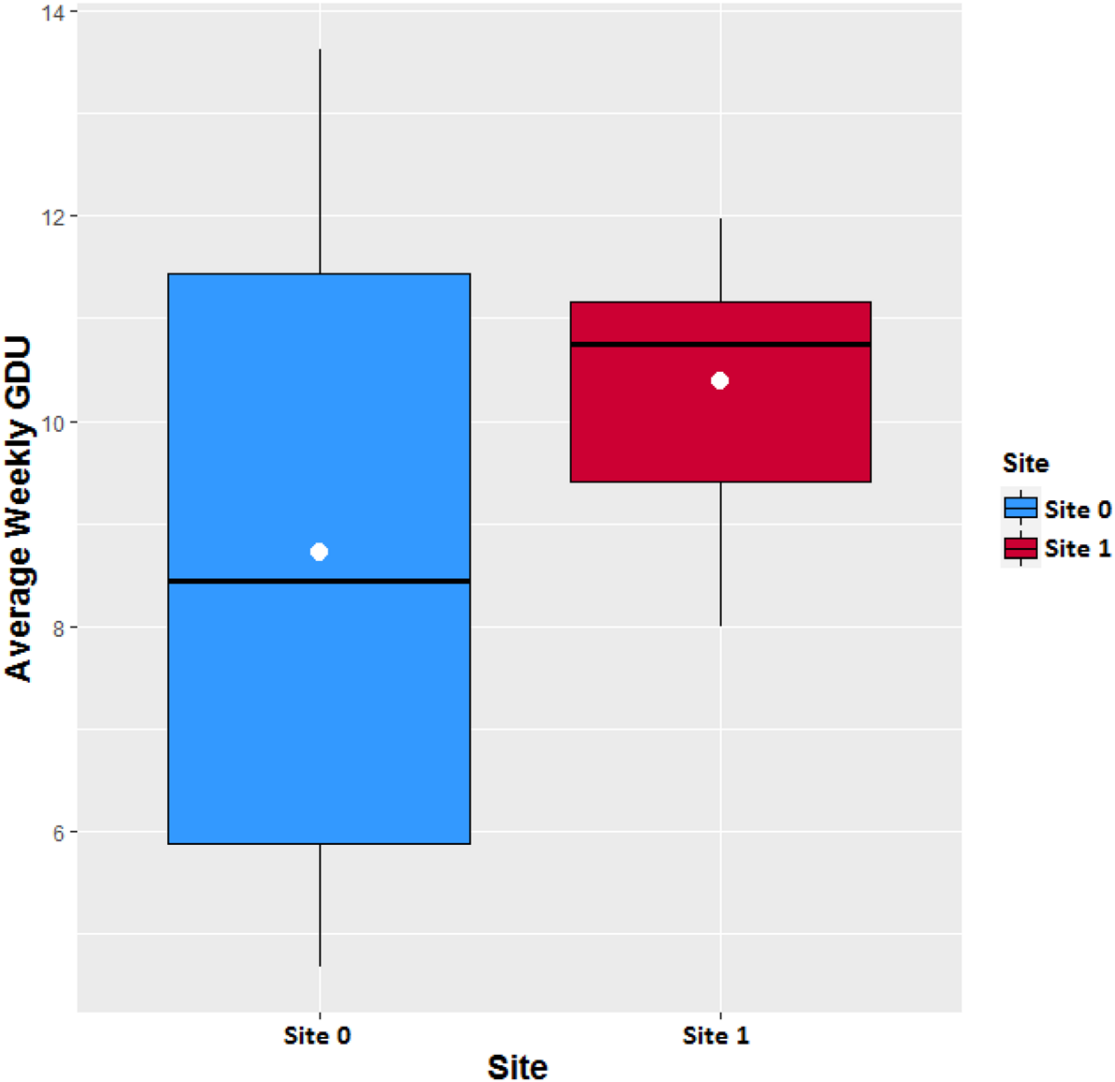
Box plot of the average weekly GDU during the last 10 years from 2010 to 2019 of each site. The white circles in each boxplots are the mean GDUs.

**Figure 2:**
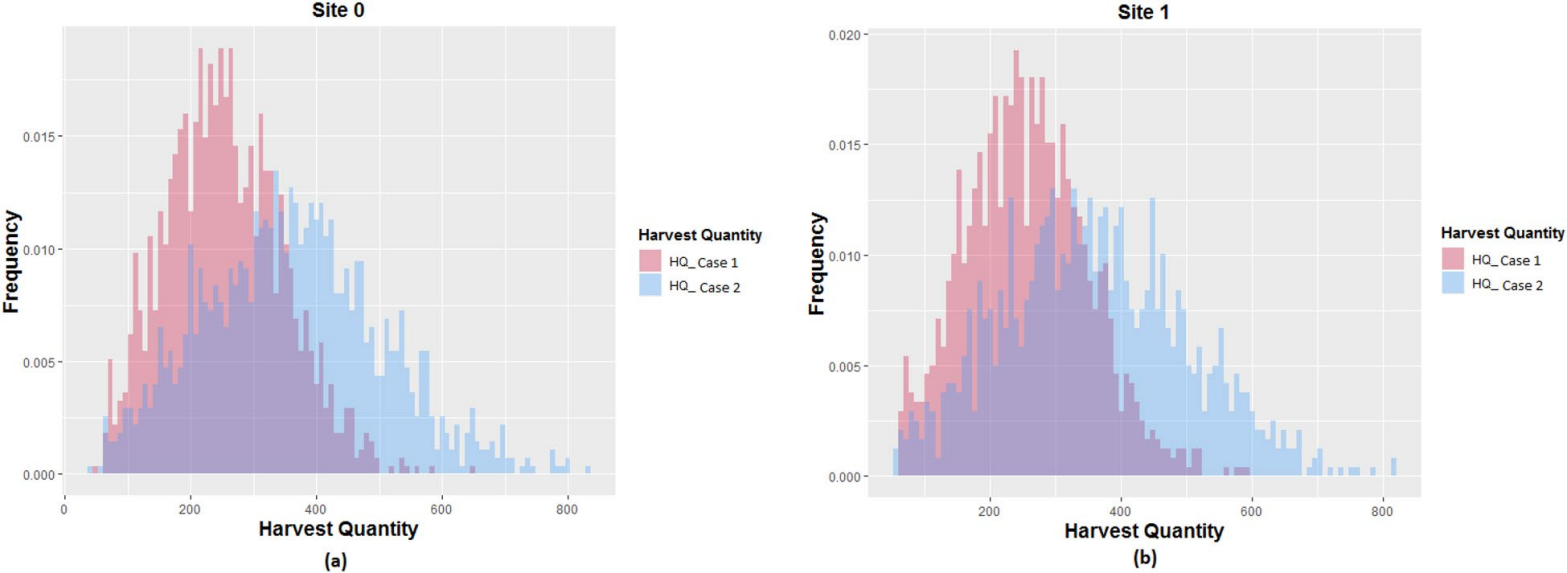
Distributions of total harvest quantity over the growing season for (**a**) 1375 seed populations planted in site 0, and (**b**) 1194 seed populations planted in site 1 for storage capacity cases 1 and 2.

The dataset contained information of two separate groups of corn hybrids including 1375 and 1194 different corn seed populations planted in site 0 and site 1, respectively. The earliest and latest planting dates (planting windows) corresponding to each seed population were provided to make sure that each hybrid is planted within its planting window. Moreover, the original planting dates which are actual planting dates of the corn hybrids were provided as a benchmark. The given data also included the GDUs in Celsius for each site for each day from 2010 to 2019. Figure 1 shows the boxplots of the average weekly GDU of site 0 and site 1 from 2010 to 2019. We observe that site 0 has a lower median GUD than the lower quartile of site 1. As a result, crops at site 0 are expected to take longer than site 1 to accumulate necessary GDU to reach full maturity. Harvest quantities of these seed populations were provided for two storage capacity cases and their distributions are shown in Fig. 2 for site 0 and site 1. It can be seen that case 2 requires a higher storage capacity than case 1 for both sites.

## Methods

### Data preprocessing

In order to balance complexity and accuracy, we decided to model the timeline of crop growth on a weekly basis. As a result, we converted the early and late planting dates to their corresponding week numbers using the Microsoft Excel WEEKNUM function, where week 1 begins on January 1, and all subsequent weeks begin on Sundays.

**Figure 3:**
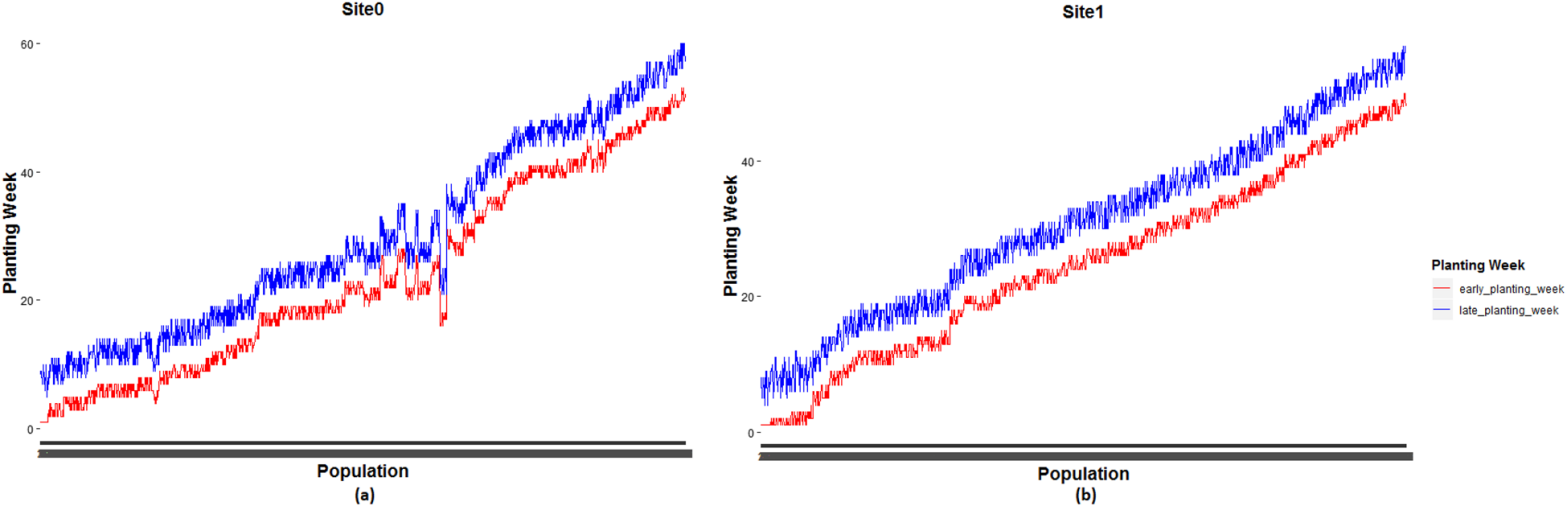
Weekly planting windows of (**a**) 1375 seed populations planted in site 0 and (**b**) 1194 seed populations planted in site 1.

Figure 3 shows the weekly planting window of each of 1375 and 1194 populations planted in site 0 and site 1, respectively.

### GDU prediction

In order to predict weekly GDUs for the 70 weeks after January 1st, 2020 using historical daily GDUs from 2010 to 2019, we designed a recurrent neural network (RNN) model, since RNN models can capture temporal dependencies. Long short-term memory (LSTM)^17^ model is a type of recurrent neural network, which can capture long-term time dependencies in the the data without having problems such as vanishing gradients. An LSTM unit is usually composed of cell, an input gate, an output gate and a forget gate which control the flow of information through time steps. We considered the following three versions of LSTM models to determine the best model structure for the two sites.

#### Vanilla RNN

Vanilla RNN is a basic version of recurrent neural network where they use their internal hidden state units (memory) to capture the temporal effects of data.

#### Bidirectional LSTM

Bidirectional LSTMs are an extension of traditional LSTMs where they process the temporal information in both forward and backward directions.

#### Stacked LSTM

We used two hidden layers each with 50 LSTM units each using ReLU activation function. The model used Adam optimizer and stochastic gradient descent to optimize the mean squared error (MSE) loss function.

These three LSTM models were trained using all weekly GDUs from 2010 to 2019. Let $G_y^w$ denote the GDU of week *w* in year *y* with $\forall w \in \{1, ..., 52\}$ and $\forall y\in \{2010, ..., 2019\}$. The LSTM model explains the GDU variable $G_y^w$ as a response of *k* previous years in the same week: $\bigg \{G_{y-k}^w, G_{y-k+1}^w, G_{y-k+2}^w, G_{y-k+3}^w,.....,G_{y-1}^w \bigg \}$. We considered three periodic lags including 3, 4, and 5 and found that 3 years to yield the best results. As such, we used the 312 weekly GDUs from 2010 to 2018 as the training data and the 52 weekly GDUs in 2019 as the validation data to compare the aforementioned LSTM models. We used root mean square error (RMSE), mean absolute error (MAE), and correlation coefficient as comparison criteria. Results are shown in Table 1, which suggest that Stacked LSTM and Bidirectional LSTM with 3 years periodic lag had the best performance for site 0 and site 1, respectively.

**Table 1:** Comparison of predictive performances of three LSTM models for two sites.

Site	Method	Lag	RMSE	MAE	Correlation coefficient(%)	Number of samples
Site0	Vanilla LSTM	3	7.21	6.06	0.98	312
		4	8.30	7.25	0.97	260
		5	7.92	6.59	0.98	208
	Bidirectional LSTM	3	9.76	6.82	0.93	312
		4	8.01	6.97	0.98	260
		5	9.85	8.73	0.98	208
	Stacked LSTM	**3**	**7.07**	**5.91**	**0.98**	**312**
		4	8.45	7.29	0.97	260
		5	7.77	6.34	0.97	208
Site1	Vanilla LSTM	3	5.43	4.24	0.83	312
		4	6.37	4.72	0.81	260
		5	6.47	4.46	0.84	208
	Bidirectional LSTM	**3**	**5.19**	**3.93**	**0.83**	**312**
		4	6.54	4.57	0.80	260
		5	5.63	4.30	0.83	208
	Stacked LSTM	3	6.24	5.18	0.83	312
		4	5.49	4.02	0.82	260
		5	6.77	5.56	0.83	208

The best results are in bold.

**Figure 4:**
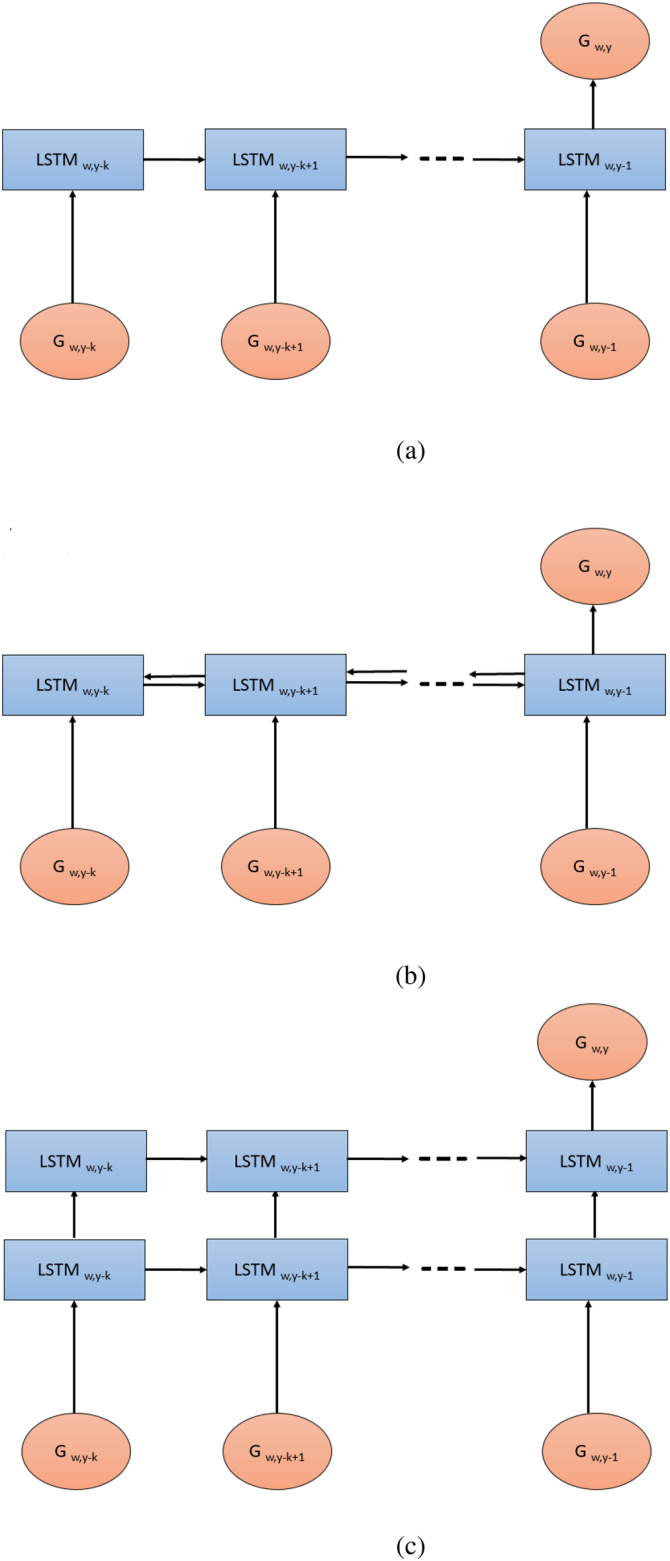
Three LSTM models for GDU prediction with a *k*-year lag. Subfigures (**a–c**) are for vanilla, bidirectional, and stacked LSTMs, respectively.

The Stacked and Bidirectional LSTM networks were then trained again to predict $G_y^w$ for the year 2020 using weekly GDU data from 2017 to 2019. Finally, we used the predicted 2020 data and historical data of 2018 and 2019 to predict weekly GDU in 2021 for the first 20 weeks. The structures of LSTM networks are illustrated in Fig. 4.

### Optimization models

In this section we propose four optimization models for scheduling planting and harvesting dates of seed populations. The first two models are for case 1, in which storage capacities for sites 0 and 1 are given as 7000 and 6000 ears, respectively. The first model is a deterministic one, considering a single GDU scenario with predicted weekly GDU values, and the second model is a stochastic one, considering ten historical years of weekly GDU data as ten scenarios. The third and forth models are, respectively, deterministic and stochastic models for case 2, in which storage capacities for the two sites are decision variables.

#### Deterministic model for case 1

In this model, predicted weekly GDUs from “GDU prediction” section were used. This model consists of the following decision variables:

2 $$\begin{aligned} t_{ij}^\text {p}= & {} {\left\{ \begin{array}{ll} 1, &{} \text {if population } \; i\;  \text {is planted in week } j\\ 0, &{} \text {otherwise} \end{array}\right. }, \end{aligned}$$

3 $$\begin{aligned} t_{ij}^\text {h}= & {} {\left\{ \begin{array}{ll} 1, &{} \text {if population} \, i  \; \text {is harvested in week } j\\ 0, &{} \text {otherwise}\\ \end{array}\right. }, \end{aligned}$$

4 $$\begin{aligned} w_j= & {} {\left\{ \begin{array}{ll} 1, &{} \text {if any population is harvested in week } j\\ 0, &{} \text {otherwise}\\ \end{array}\right. }. \end{aligned}$$

Parameters of this model include:

- *C*: storage capacity of a site.
- $GDU_j$: cumulative weekly GDU from week 1 to week *j*.
- *N*: total number of populations.
- *T*: number of weeks after January 1st of planning year. In the 2021 Syngenta crop challenge, $T=70$.
- $HQ_i$: harvest quantity (number of ears) of population *i*.
- $T^{p_i}=\{\, [l_i, u_i] \, \,\}$: the planting window for the population *i*, where $l_i$ and $u_i$ are the corresponding earliest and latest planting dates, respectively.
- $G_i^\text {min}$: number of GDUs needed by population *i* before harvesting.

We formulate our optimization model as the following:

5 $$\begin{aligned}{} & {} \min _{t_{ij}^\text {p},t_{ij}^{\text {h}},w_j} \quad \sum _{j=1}^{T} |w_j C-\sum _{i=1}^{N} HQ_i t_{ij}^{\text {h}}|, \end{aligned}$$

6 $$\begin{aligned}{} & {} s.t. \sum _{j\in T^{p_i}} t_{ij}^\text {p}=1 \quad \forall i \in \{1,...,N\}, \end{aligned}$$

7 $$\begin{aligned}{} & {} \sum _{j \not \in T^{p_i}} t_{ij}^\text {p}=0 \quad \forall i \in \{1,...,N\}, \end{aligned}$$

8 $$\begin{aligned}{} & {} \sum _{j=1}^T t_{ij}^{\text {h}}=1 \quad \forall i \in \{1,...,N\}, \end{aligned}$$

9 $$\begin{aligned}{} & {} \sum _{j=1}^T (t_{ij}^{\text {h}} \; GDU_j - t_{ij}^\text {p} \; GDU_j) \;\ge \; G_{i}^{\textit{min}} \quad \forall i \in \{1,...,N\}, \end{aligned}$$

10 $$\begin{aligned}{} & {} \sum _{j=1}^T (t_{ij}^{\text {h}} \; GDU_{j-1} - t_{ij}^\text {p} \; GDU_j) \le \; G_{i}^{\textit{min}} -1 \quad \forall i \in \{1,...,N\}, \end{aligned}$$

11 $$\begin{aligned}{} & {} N\, w_j \;\ge \; \sum _{i=1}^{N} t_{ij}^{\text {h} } \quad \forall j \in \{1,...,T\}, \end{aligned}$$

12 $$\begin{aligned}{} & {} t_{ij}^\text {p},\, t_{ij}^{\text {h} },\, w_j \in \{0,1\} \quad \forall i \in \{1,...,N\},\quad \forall j \in \{1,...,T\}. \end{aligned}$$

Here, the objective (5) is to minimize the difference between the weekly harvest quantity and the capacity for each harvesting week while using the minimum number of weeks for harvesting. We adopt the absolute value function in our objective function because it can be easily linearized and is computationally more tractable^18^. Constraints (6) and (7) make sure that each population is planted within its corresponding planting window. Constraint (8) means that each population can only be harvested in one week. Constraints (9) and (10) enforce the model to harvest populations as soon as they accumulate their required GDU. Constraint (11) requires that $w_j=1$ when any population is harvested in week *j*. Finally, constraint (12) defines all decision variables as binary.

Due to having the absolute value function inside our objective, the above-mentioned model is a nonlinear optimization problem which is hard to solve. As a result, we reformulate our model into an equivalent mixed integer linear programming (MILP) by introducing a set of new variables which is as follows^18^:

13 $$\begin{aligned}{} & {} \min _{t_{ij}^\text {p}, t_{ij}^\text {h}, w_j, e_{j}^{+}, e_{j}^{-}} \;\;\sum _{j=1}^{T} (e_{j}^{+}+e_{j}^{-}), \end{aligned}$$

14 $$\begin{aligned}{} & {} w_jC-\sum _{i=1}^{N} HQ_i \, t_{ij}^\text {h} =e_{j}^{+}-e_{j}^{-} \; \; \; \;\;\forall j \in \{1,...,T\}, \end{aligned}$$

15 $$\begin{aligned}{} & {} \text {Constraints } (6)-(11), \end{aligned}$$

16 $$\begin{aligned}{} & {} t_{ij}^\text {p},\, t_{ij}^\text {h},\, w_j \in \{0,1\},\,\, e_{j}^{+}, e_{j}^{-} \ge 0 \; \; \; \;\;\forall i \in \{1,...,N\},\, \forall j \in \{1,...,T\}. \end{aligned}$$

Here, the objective (13) is to minimize the positive and negative errors between the storage capacity and the sum of harvested quantities for each harvesting week which is equivalent to objective (5). In constraints (14) and (15) we define the two error terms $e_{j}^{+}$ and $e_{j}^{-}$. Because in this problem the goal is to have weekly harvest quantities under the capacity, we put $e_{j}^{-}$ zero in the optimal solution. As a result, the model will be improved by reducing the positive error. Constraint (16) indicates the appropriate types of the decision variables.

#### Stochastic model for case 1

Considering multiple GDU scenarios during the last 10 years from 2010 to 2019 together, we now propose a more generalized optimization model to find the optimal planting dates of all populations for all 10 historical GDU scenarios so that based on the weekly GDU values of each of ten GDU scenarios the corn populations will be harvested on different dates. Then the maximum weekly harvest quantites among all 10 GDU scenarios will be consistent and below the capacity of the site. The decision variables of the generalized optimization model are:

17 $$\begin{aligned} t_{ij}^{\text {p}}= & {} {\left\{ \begin{array}{ll} 1, &{} \text {if population } \; i \text {is planted in week } j\\ 0, &{} \text {otherwise} \end{array}\right. }, \end{aligned}$$

18 $$\begin{aligned} t_{ij}^{\text {h}k}= & {} {\left\{ \begin{array}{ll} 1, &{} \text {if population } \; i \text {is harvested in week } j \text {with GDU of year} k\\ 0, &{} \text {otherwise}\\ \end{array}\right. }. \end{aligned}$$

This optimization model consists of the following notations:

- *k*: index of the year corresponding to different GDU scenarios from 2010 to 2019 of each site.
- *C*: the storage capacity of each site.
- $A_{jk}$: the harvest quantity of week *j* of GDU scenario of year *k*.
- *K*: number of years which is 10.
- $A_{j\text {max}}$: the maximum weekly harvest quantity among all GDU scenarios of *K* years.
- $GDU^{k}_j$: the cumulative weekly GDU of year *k* which is accumulated till week *j*.
- *N*: the total number of populations.
- *T*: number of weeks after Jan 1st of planning year (it is 70 weeks for the 2021 Syngenta crop challenge).
- $HQ_i$: number of ears (harvest quantity) of case 1 produced by population *i*.
- $T^{p_i}=\{\, [l_i, u_i] \, \,\}$: the planting window for the population *i*, where $l_i$ and $u_i$ are the corresponding earliest and latest planting dates, respectively.
- $G_i^\text {min}$: number of growing degree units needed before harvesting population *i* (required GDUs for population *i*).

As such, we define our more generalized optimization model to consider all GDU scenarios and find one set of optimal planting dates which works for all GDU scenarios:

19 $$\begin{aligned}{} & {} \min _{t_{ij}^\text {p},t_{ij}^{\text {h}k},w_j^{k}} \; \sum _{j=1}^{T} |C-A_{j\text {max}}|, \end{aligned}$$

20 $$\begin{aligned}{} & {} A_{jk} \;=\; \sum _{i=1}^{N} HQ_i \, t_{ij}^{\text {h} k} \; \; \; \;\;\forall j \in \{1,...,T\} \; \; \; \;\;\forall k \in \{1,...,K\}, \end{aligned}$$

21 $$\begin{aligned}{} & {} A_{j\text {max}} \;=\; max_k(A_{jk})\; \; \; \;\;\forall j \in \{1,...,T\} , \end{aligned}$$

22 $$\begin{aligned}{} & {} s.t. \sum _{j\in T^{p_i}} t_{ij}^\text {p}=1 \; \; \; \;\;\forall i \in \{1,...,N\}, \end{aligned}$$

23 $$\begin{aligned}{} & {} \sum _{j \not \in T^{p_i}} t_{ij}^\text {p}=0 \; \; \; \;\;\forall i \in \{1,...,N\}, \end{aligned}$$

24 $$\begin{aligned}{} & {} \sum _{j=1}^T t_{ij}^{\text {h}k}=1 \; \; \; \;\;\forall i \in \{1,...,N\} \; \; \; \;\;\forall k \in \{1,...,K\}, \end{aligned}$$

25 $$\begin{aligned}{} & {} \sum _{j=1}^T t_{ij}^{\text {h}k} \; GDU_j^{k} - t_{ij}^\text {p} \; GDU_j^{k} \;=\; G_{i}^{\textit{min}} \; \; \; \;\;\forall i \in \{1,...,N\} \; \; \; \;\;\forall k \in \{1,...,K\} , \end{aligned}$$

26 $$\begin{aligned}{} & {} t_{ij}^\text {p},\, t_{ij}^{\text {h} k} \in \{0,1\} \; \; \; \;\;\forall i \in \{1,...,N\},\, \forall j \in \{1,...,T\}\; \; \; \;\;\forall k \in \{1,...,K\}. \end{aligned}$$

The objective function (19) is to minimize the sum of differences between maximum weekly harvest quantities among 10 GDU scenarios and the capacity of the site. The key to solving model (19) is $t_{ij}^\text {p}$ that represents the planting dates of the populations. Once this variable is revealed, then given each GDU scenario’s weekly GDU values the harvesting weeks will be known. It is because the populations should be harvested as soon as they reach their required GDUs. So, given the planting schedule of corn populations because of the different weekly GDUs for each GDU scenario the harvesting week of populations and subsequently weekly harvest quantities would be different for each GDU scenario.

#### Heuristic algorithm

Unlike the optimization model (13)–(16) proposed in “Deterministic model for case 1” section which can be solved using the existing branch-and-bound algorithms^19^, solving the optimization model (19)–(26) using existing algorithms and solvers is extremely time-consuming due to numerous number of variables. Therefore, this section presents a metaheuristic algorithm, simulated annealing (SA)^20^, for solving the corn scheduling problem considering multiple GDU scenarios together (19)–(26), which is computationally tractable and searches for a local optimal solution to model (19)–(26). Simulated annealing is a probabilistic optimization method designed for finding the global minimum of a cost function that may possess several local minima. SA algorithm is based on the emulation of physical annealing process where a heated solid is cooled down to reach a minimum energy configuration^21^. Our goal is to find an optimal planting date, $t^\text {p*}$, considering GDU scenarios of 10 different years which minimizes the objective function defined in Eq. (19). In our problem definition, we no longer need to include the harvest dates variables in our model because of the assumption of harvesting corn populations as soon as they accumulate their respective minimum required GDUs. Our heuristic algorithm starts with a random solution ($x_0$) and initial temperature ($T_0$), and continues until a maximum of $k_{max}$ iterations. Steps of the heuristic algorithm used in this study are as follows:
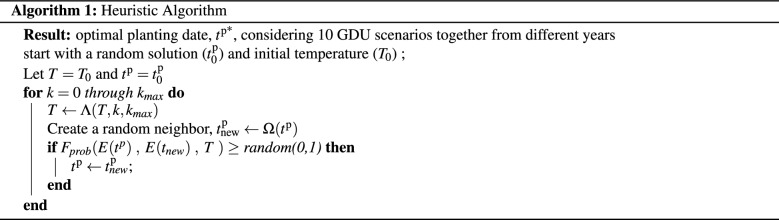


Here, $\Lambda (T,k,k_{max})$, $\Omega (t^\text {p})$, and $F_{prob}(E(t^\text {p})\;,\;E(t_{\text {new}})\;,\;T \;)$ are temperature, create-neighbor, and energy functions, respectively. We used the following temperature function to decay the initial temperature ($T_0$) during the heuristic algorithm process: $\Lambda (T,k,k_{max})=T_0\,\alpha ^k, \; \text {where}\; \;k\le k_{max}, \; \text {and}\;\; 0\le \alpha \le 1.$ Higher temperature allows more exploration of the solution space at the beginning of the optimization process. We set max iteration, decay rate ($\alpha$), and initial temperature to be 700, 0.995, and 30,000, respectively. We designed a create-neighbor function, $\Omega (t^\text {p})$, which creates a new solution based on the previous solution which considers both the exploitation of current solution and exploration of solution space. As such, $\Omega (t^\text {p})$ first finds the weeks which have the maximum and minimum harvest quantities across all GDU scenarios. For the populations at the maximum harvest quantity week, we select three populations with minimum harvest quantities and randomly move either forward or backward their respective planting dates one or two weeks. For the week with minimum harvest quantity, we try to move populations from closest weeks with the larger harvest quantities to the week with minimum harvest quantity. We used the following energy function to compute the probability of the acceptance of the current solution: $F_{prob}(E(t^\text {p})\;,\;E(t_{\text {new}})\;,\;T \;)=e^{-(E(t^\text {p})-E(t_{\text {new}}))/T},\;$ where $E(t^\text {p})$, $E(t_{\text {new}})$, and *T* are the cost of best solution so far, the cost of new solution, and the current temperature, respectively.

#### Deterministic model for case 2

This subsection illustrates our proposed optimization model for case 2 where there is not a predefined capacity, and the goal is to determine planting and harvesting dates of each population (during *T* weeks) and also the lowest capacity required for each site. The optimization model for case 2 has the same decision variables and constraints with the optimization model proposed for case 1, but the objective function is changed to achieve the goal of case 2. Here $\theta _{w}$ is a coefficient for the number of harvesting weeks, and it is set to be 1. The optimization model for case 2 is as follows:

27 $$\begin{aligned}{} & {} \min \;\; \max \;\; \{\sum _{i=1}^{N} HQ_i \, t_{i1}^\text {h}, \sum _{i=1}^{N} HQ_i \, t_{i2}^\text {h},..., \sum _{i=1}^{N} HQ_i \, t_{iT}^\text {h}\} +\theta _{w}\sum _{j=1}^{T} w_j, \end{aligned}$$

28 $$\begin{aligned}{} & {} \text {Constraints } (6)-(10), (14), \end{aligned}$$

29 $$\begin{aligned}{} & {} t_{ij}^\text {p},\, t_{ij}^\text {h},\, w_j \in \{0,1\},\,\, e_{j}^{+}, e_{j}^{-} \ge 0 \; \; \; \;\;\forall i \in \{1,...,N\},\, \forall j \in \{1,...,T\}. \end{aligned}$$

This is a Minimax Linear Programming Problem (MLPP), and the objective (27) is to minimize the maximum amount of weekly harvest quantities which simultaneously minimizes the amount of weekly harvest quantities for all weeks while using the minimum number of harvesting weeks. Since the objective (27) is nonlinear, we reformulate our model into an equivalent mixed integer linear programming (MILP) by introducing a new variable denoted by *z*^22^:

30 $$\begin{aligned}{} & {} \min \; \; z +\theta _{w}\sum _{j=1}^{T} w_j, \end{aligned}$$

31 $$\begin{aligned}{} & {} z \;\ge \; \sum _{i=1}^{N} HQ_i \, t_{ij}^\text {h} \; \; \; \;\;\forall j \in \{1,...,T\}, \end{aligned}$$

32 $$\begin{aligned}{} & {} \text {Constraints} \; \;\; \; \;\; (6)-(10), (14), \end{aligned}$$

33 $$\begin{aligned}{} & {} t_{ij}^\text {p},\, t_{ij}^\text {h},\, w_j \in \{0,1\},\,\, e_{j}^{+}, e_{j}^{-}, z \ge 0 \; \; \; \;\;\forall i \in \{1,...,N\},\, \forall j \in \{1,...,T\}. \end{aligned}$$

Here, the objective is to minimize the summation of the maximum value of the weekly harvest quantities and the total number of harvesting weeks. Constraint (31) ensures that the maximum value of the weekly harvest quantities is always greater than or equal to the amount of harvest quantity of each week.

#### Stochastic model for case 2

As it was mentioned previously, in case 2, there is not a predefined capacity for each site and we need to determine the lowest capacity required. This subsection presents a more generalized optimization model which considers all ten GDU scenarios together to determine the optimal planting dates of all populations. The optimal planting dates of all populations will result in different harvesting dates based on the weekly GDU values of each of ten GDU scenarios. The goal here is to determine the lowest capacity required in a way that the maximum weekly harvest quantities among all 10 GDU scenarios will be consistent.

To this end, we define a new loss function which computes the sum of the absolute differences between each weekly harvest quantity which is the maximum weekly harvest quantity among all GDU scenarios and its neighboring weekly harvest quantities. Minimizing this loss function ensures consistent harvest quantities with lowest possible storage capacity. We use the same heuristic algorithm described in “Heuristic algorithm” section.

## Results

In this section, we present the quantitative results of our optimization models for case 1 and case 2 considering predicted GDU scenario, and all 10 GDU scenarios together. In the 2021 Syngenta crop challenge, participants were asked to schedule the planting date of each seed population sometimes within the given planting window for each seed population and the harvesting dates during 70 weeks after January 1st 2020. As it was discussed in “GDU prediction” section the daily GDUs of these 70 weeks were unknown a priori and as a part of the challenge we made use of historical daily GDUs from 2010 to 2019 provided for each site to estimate GDUs of these 70 weeks for each site which is the predicted GDU scenario. We also solved the optimization models considering all 10 historical GDUs together.

We implemented our MILP models (13)–(16) and (30)–(33) for case 1 and case 2 respectively considering predicted GDU scenario in MATLAB R2018a and solved with MILP commercial solver Gurobi Optimizer. The heuristic algorithm considering multiple GDU scenarios was implemented in Python for both cases 1 and 2. A summary of results from the deterministic and stochastic models for storage capacity cases 1 and 2 for both sites is provided in Table 2.

**Table 2:** A summary of results from the deterministic and stochastic models for storage capacity cases 1 and 2 for both sites.

Site number-capacity case	GDU scenario	Optimization model	Difference between harvest quantities and capacity for original planting dates	Difference between harvest quantities and capacity for optimal planting dates	Lowest storage capacity for original planting dates	Lowest storage capacity for optimal planting dates
Site 0-capacity case 1	Deterministic	MILP model	223,373	6,793	−	−
Site 1-capacity case 1	Deterministic	MILP model	116,153	4,661	−	−
Site 0-capacity case 1	Stochastic	Both MILP model and heuristic algorithm are infeasible	−	−	−	−
Site 1-capacity case 1	Stochastic	Heuristic algorithm	172,851	87,421	−	−
Site 0-capacity case 2	Deterministic	MILP model	−	−	37,247	10,795
Site 1-capacity case 2	Deterministic	MILP model	−	−	16,220	8,108
Site 0-capacity case 2	Stochastic	Both MILP model and heuristic algorithm are infeasible	–	–	–	–
Site 1-capacity case 2	Stochastic	Heuristic algorithm	–	–	21,811	11,192

### Results of the deterministic model for case 1

The MILP model for case 1 (13)–(16) considering predicted GDU scenario which was calculated in “GDU prediction” section was run in MATLAB for each site. Input variables for the optimization model for each site include: the storage capacity (*C*) of 7000 ears for site 0 and 6000 ears for site 1, total number of populations (*N*) of 1375 planted in site 0 and 1194 planted in site 1, total number of ears produced by each seed population ($HQ_i$) planted in site 0 and site 1 given for case 1, and required GDUs of each seed population ($G_i^\text {min}$) planted in site 0 and site 1. The cumulative weekly GDUs ($GDU_j$) of each site are calculated using the predicted weekly GDU from “GDU prediction” section for each site. The number of weeks after Jan 1st of planning year (*T*) is equal to 70 for both sites. Finally, the planting windows of seed populations are given to the model so that the planting date for each seed population falls into its corresponding planting window ($T^{p_i}$).

**Figure 5:**
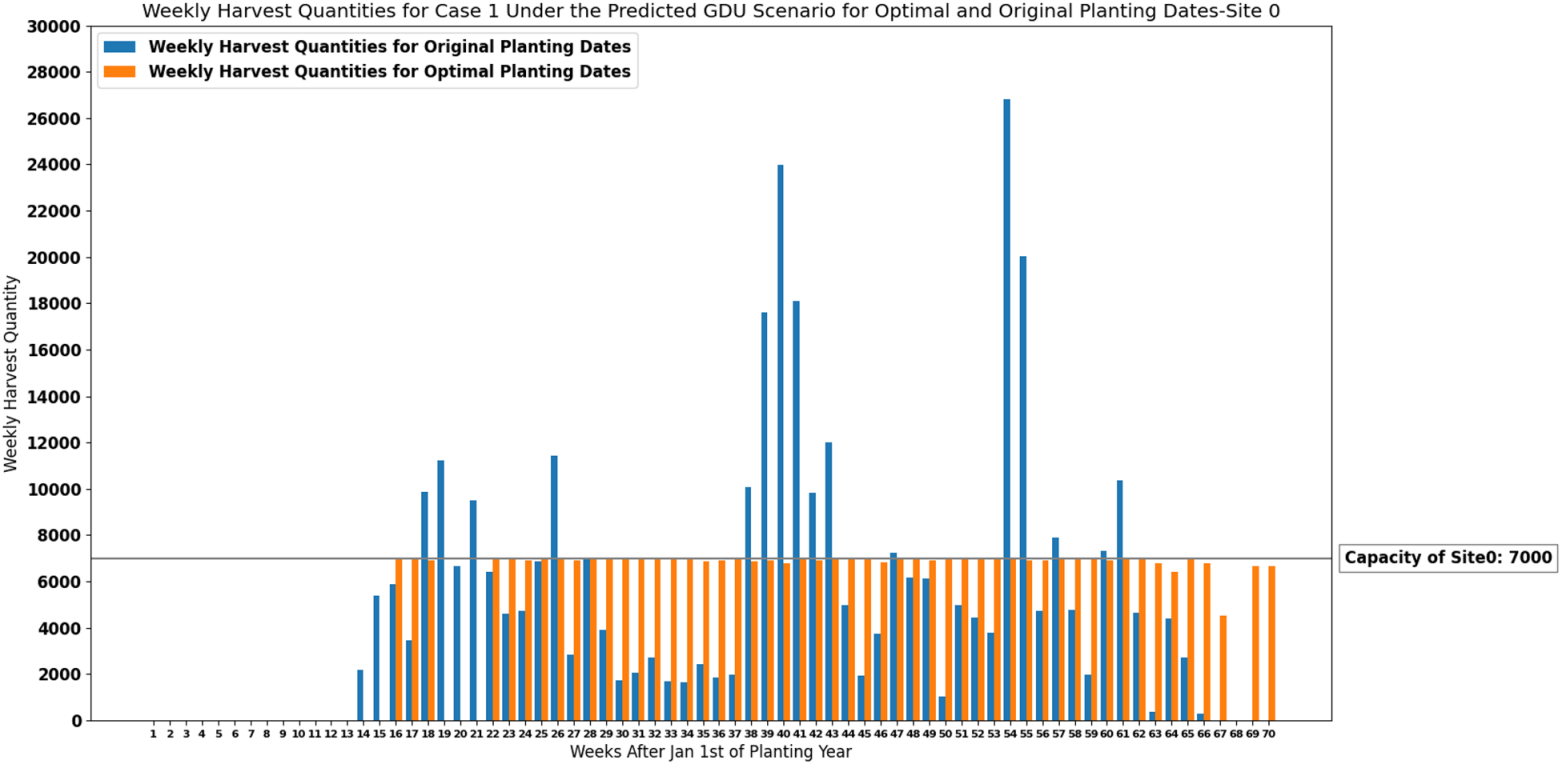
Weekly harvest quantities of site 0 with a capacity of 7000 ears for case 1 under the predicted GDU scenario for optimal and original planting dates.

**Figure 6:**
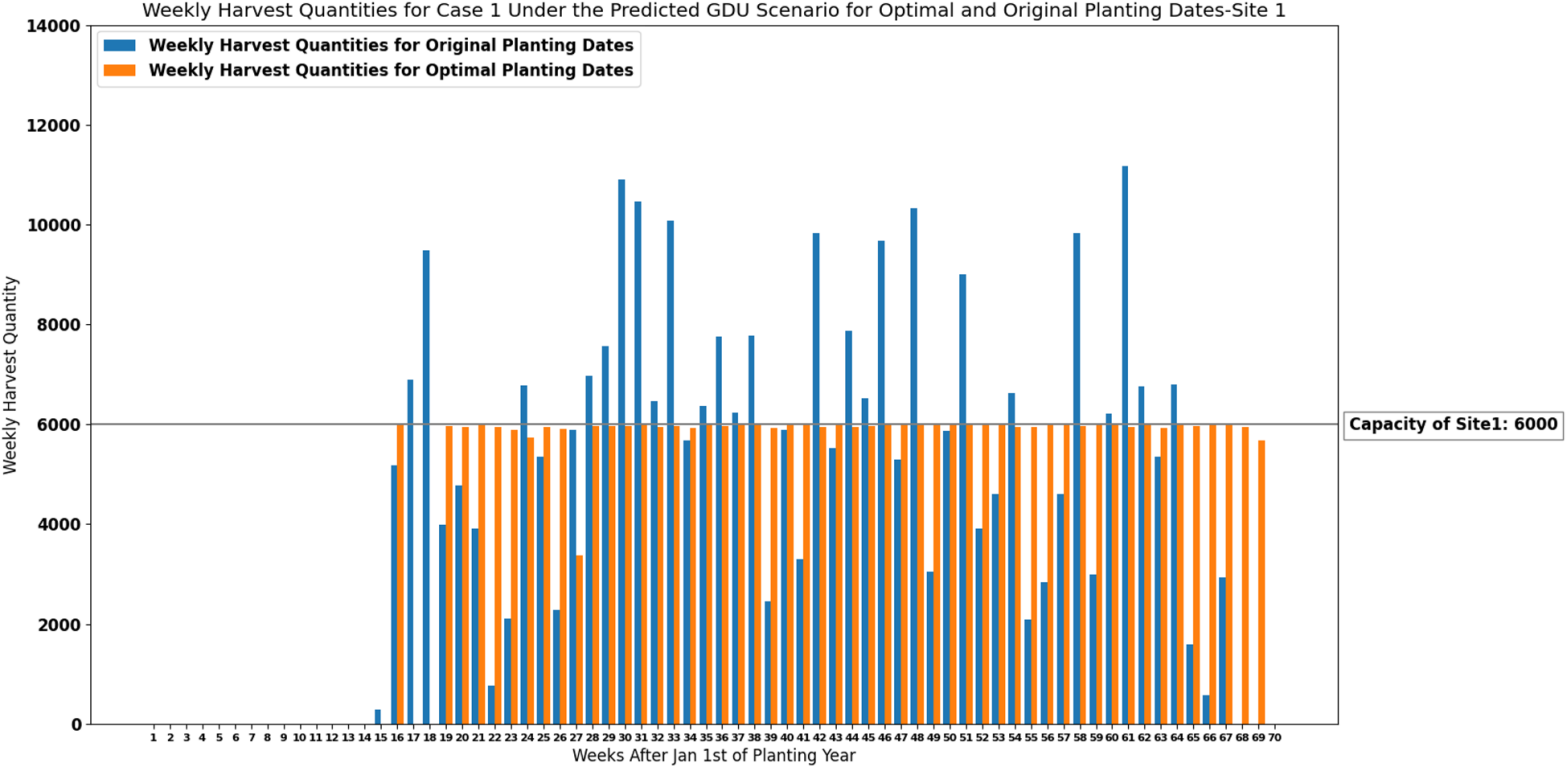
Weekly harvest quantities of site 1 with a capacity of 6000 ears for case 1 under the predicted GDU scenario for optimal and original planting dates.

Weekly harvest quantities considering the predicted GDU scenario for both optimal planting dates and original planting dates are shown in Figs. 5 and 6 for site 0 and site 1 respectively. Optimal planting dates are the optimal planting dates resulted from our proposed MILP model for case 1 (13)–(16) considering the predicted GDU scenario, and original planting dates are the actual planting dates of the populations which were given by the Syngenta Crop Challenge. These figures suggest that the proposed MILP model (13)–(16) was able to schedule the planting and harvesting dates of the whole 1375 and 1194 populations planted in site 0 and site 1 respectively with different planting windows, required GDUs, and harvest quantities in a way that resulted in consistent weekly harvest quantities that are below the storage capacities.

The absolute maximum and absolute median difference between the weekly harvest quantity and the capacity among all harvesting weeks for case 1 considering the predicted GDU scenario for optimal and original planting dates for site 0 and site 1 are shown in Table 3. Moreover, run-time, and values of both objectives including number of harvesting weeks and sum of absolute differences between the capacity and weekly harvest quantities resulted from the proposed model (13)–(16) are presented in Table 4 for both sites.

**Table 3:** The absolute median difference between the weekly harvest quantity and the capacity (Median Dif) and the absolute maximum difference between the weekly harvest quantity and the capacity (Maximum Dif) among all harvesting weeks for site 0 and site 1 for case 1 under the predicted GDU scenario.

Site	Optimal planting		Original planting	
	Median dif	Maximum dif	Median dif	Maximum dif
0	57	2471	2220	6732
1	25	2633	118	5718

**Table 4:** Run-time, and values of both objectives including number of harvesting weeks and sum of absolute differences between the capacity and weekly harvest quantities resulted from the proposed MILP model for case 1 under the predicted GDU scenario for site 0 and site 1.

Site	Run-time (s)	Number of harvesting weeks	Sum of absolute differences
0	3191	51	6793
1	65	52	4661

**Figure 7:**
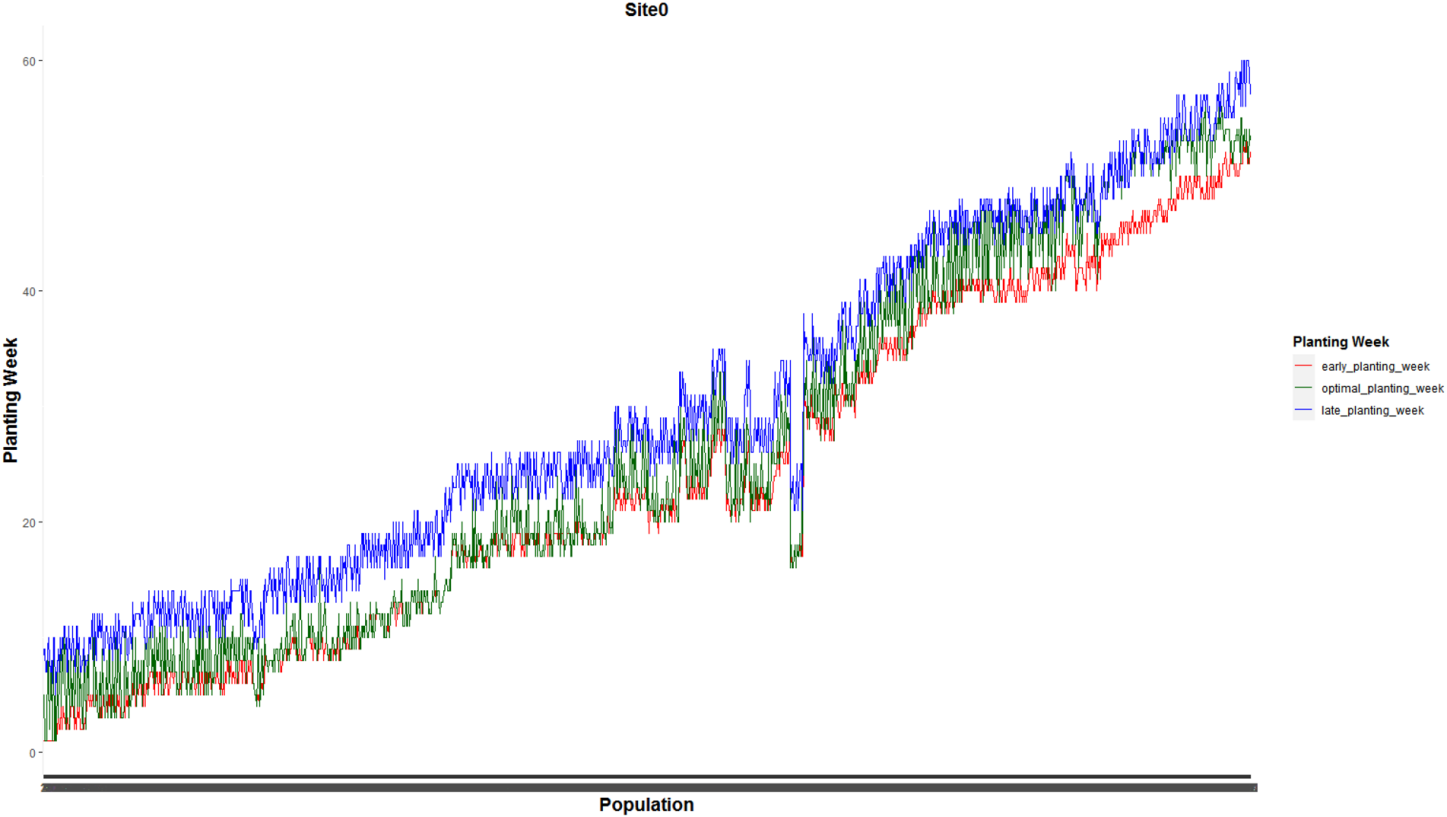
Optimal planting weeks along side with the early and late planting weeks for the the whole 1375 seed populations planted in site 0 for case 1 under the predicted GDU scenario.

**Figure 8:**
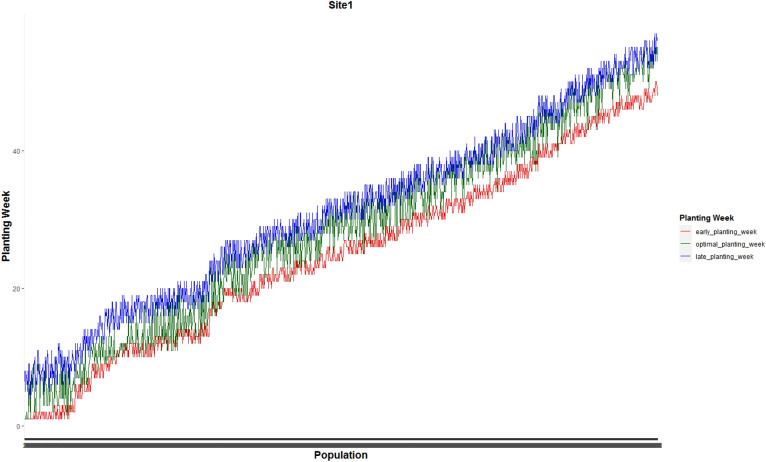
Optimal planting weeks along side with the early and late planting weeks for the whole 1194 seed populations planted in site 1 for case 1 under the predicted GDU scenario.

Figures 7 and 8 present optimal planting weeks suggested by our proposed MILP model (13)–(16) for case 1 under the predicted GDU scenario along side with the early and late planting weeks for the the whole 1375 seed populations planted in site 0 and 1194 seed populations planted in site 1 respectively. These plots show that the optimal planting date for each seed population falls into its corresponding planting window.

### Results of the stochastic model for case 1

In this subsection we present the results of our proposed heuristic algorithm for case 1. As it was discussed in “Data” section there is a lower amount of heat available for the growth of crops in site 0. As a result the growing degree units required in order for the corn population to achieve maturity accumulate slower in site 0 and the seed populations may need more than 70 weeks to be ready to be harvested. So, for site 0 all 10 GDU scenarios do not enable us to harvest the whole 1375 seed populations in 70 weeks. So, we only solved our heuristic algorithm for site 1 considering all 10 GDU scenarios for both cases 1 and 2.

**Figure 9:**
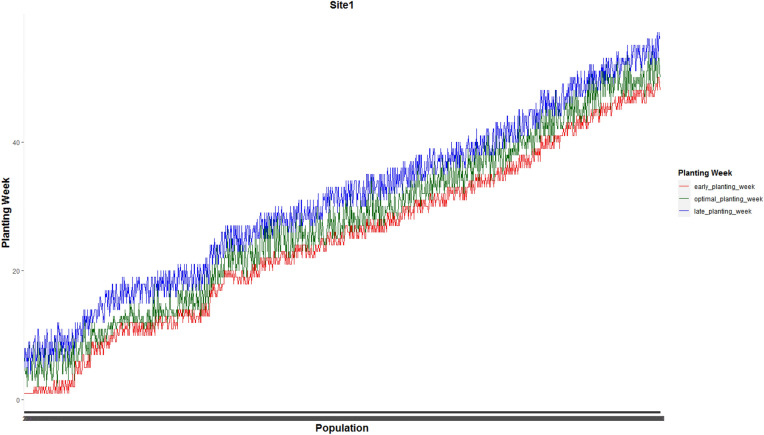
Optimal planting weeks of 1194 seed populations planted in site 1 for all 10 GDU scenarios for case 1.

The heuristic algorithm resulted in one set of planting dates shown in Fig. 9, and ten sets of harvesting dates for 10 GDU scenarios. In other words, because each seed is harvested as soon as it accumulates its required GDU, we only need to know the planting dates of the seed populations and based on each historical GDU scenario the harvesting weeks will be known.

**Figure 10:**
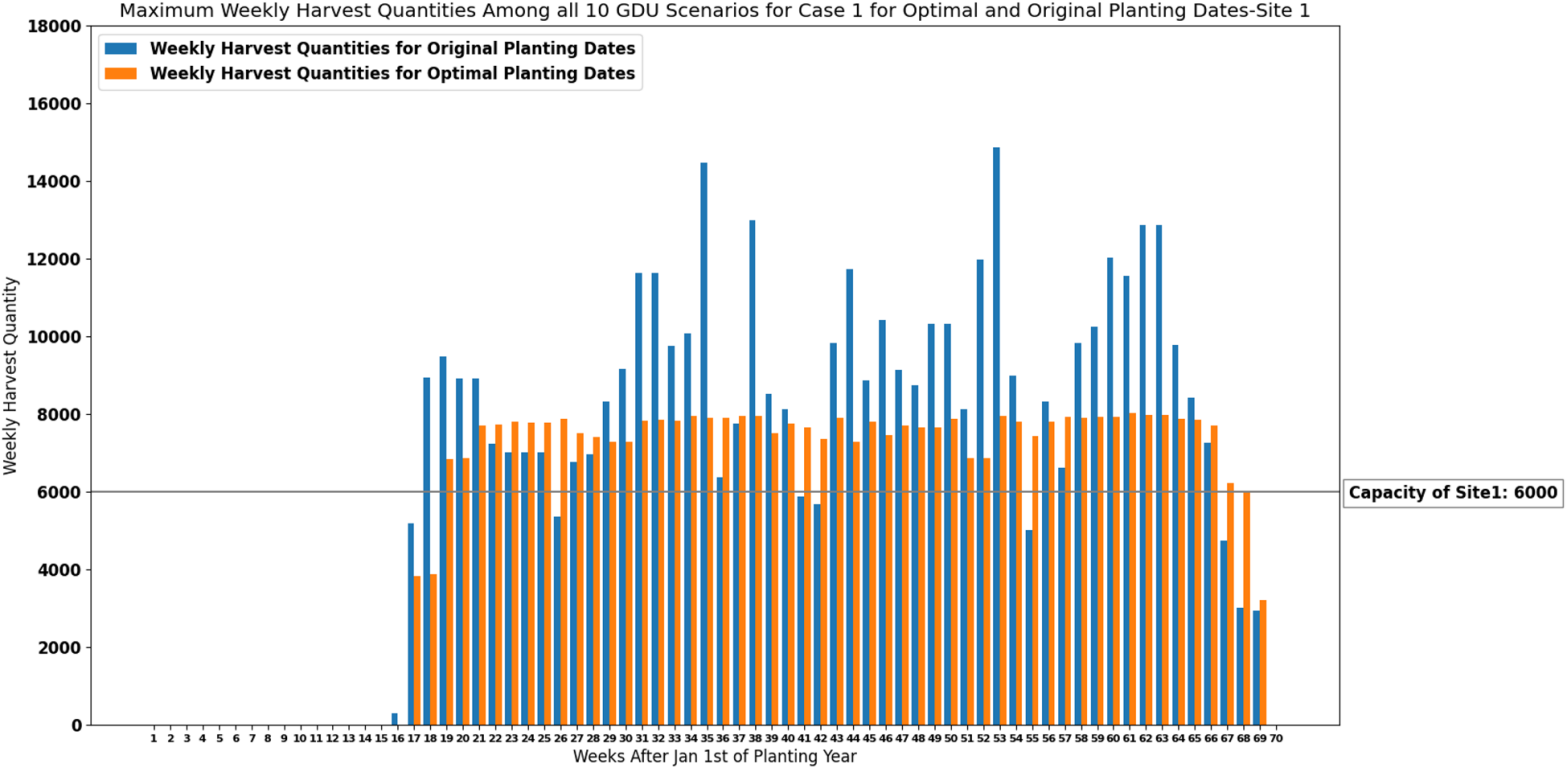
Maximum weekly harvest quantities among all 10 GDU scenarios of site 1 with a capacity of 6000 ears for case 1 for optimal and original planting dates.

Maximum weekly harvest quantities among all 10 GDU scenarios for optimal planting dates resulted from our heuristic algorithm (“Heuristic algorithm” section) and for original planting dates are shown in Fig. 10 for site 1. As it was explained in “Heuristic algorithm” section, the heuristic algorithm finds optimal planting dates by moving planting dates of populations with minimum harvest quantities at the week with maximum harvest quantity either forward or backward. For the week with the minimum harvest quantity, the model tries to move populations from closest weeks with the larger harvest quantities to the week with minimum harvest quantity. As it is shown in Fig. 10, weeks 17, 18, and 69 are the weeks with the minimum harvest quantities and they are causing an inconsistency in weekly harvest quantities. In Table 5, we manually tried to move seed populations with the minimum harvest quantities from taller bars to shorter bars to check whether the model could have improved the results. For example, the planting date of population ID 94 corresponding to week 19 was changed from week 3 to 2, and it not only did not change the weekly harvest quantities of weeks 17 and 18 but also caused inconsistency by increasing the difference between weeks 19 and 20. Moreover, it was impossible to change the planting date of population ID 1061 corresponding to week 68 from week 52 to 53 because week 52 is the last day of its planting window. These results from Table 5 suggest that the planting dates resulted from our heuristic algorithm are reasonable since the changes could not improve the results.

**Table 5:** Information of population IDs 94 and 1061 with the lowest harvest quantities among all populations corresponding to target weeks 19 and 68 respectively and how changing their planting dates to other weeks can affect the results.

Target week	19	68
Population ID	94	1061
Early planting week	2	43
Late planting week	9	52
Harvest quantity	160	68
Optimal planting week	3	52
New planting week	2	Not possible
Optimal HQs for weeks of 17/18/19/20	3826/3881/**6845**/6869	Not applicable
HQs after change of planting week for weeks of 17/18/19/20	3826/3881/**6685**/6869	Not applicable

Weekly harvest quantities of week 19 before and after the change are in bold.

The values of the sum of absolute differences between maximum weekly harvest quantities among all 10 GDU scenarios and the capacity resulted from optimal planting dates suggested by the objective function of the proposed heuristic algorithm and from original planting dates for site 1 are presented in Table 6. The run-time of the algorithm is also presented in Table 6.

**Table 6:** Run-time of the proposed heuristic algorithm and the values of the sum of absolute differences between maximum weekly harvest quantities among all 10 GDU scenarios and the site capacity resulted from optimal planting dates suggested by the objective function of the proposed heuristic algorithm and from original planting dates for case 1 under the multiple GDU scenarios for site 1.

Planting date	Run-time (h)	Sum of absolute differences
Optimal planting	20	87,421
Original planting	–	172,851

### Results of the deterministic model for case 2

The same input variables as the MILP model of case 1 (13)–(16) were used in the MILP model of case 2 (30)–(33) except the number of ears produced by each population ($HQ_i$) planted in site 0 and site 1. As it was discussed in “Data” section there are different quantities for the number of ears produced by each population ($HQ_i$) planted in site 0 and site 1 for this case in which the model is supposed to determine the lowest capacity required for each site. Because of the complexity of this MILP model (30)–(33) we used stopping criterion of 0.1 % optimality gap for each site.

The lowest storage capacity required for each site under the predicted GDU scenario for optimal planting dates suggested by the MILP model (30)–(33) and for original planting dates are presented in Table 7.

**Table 7:** The lowest storage capacities required for site 0 and site 1 under the predicted GDU scenario for optimal planting dates and original planting dates.

Site	Lowest capacity for optimal planting dates	Lowest capacity for original planting dates
0	10,795	37,247
1	8108	16,220

**Figure 11:**
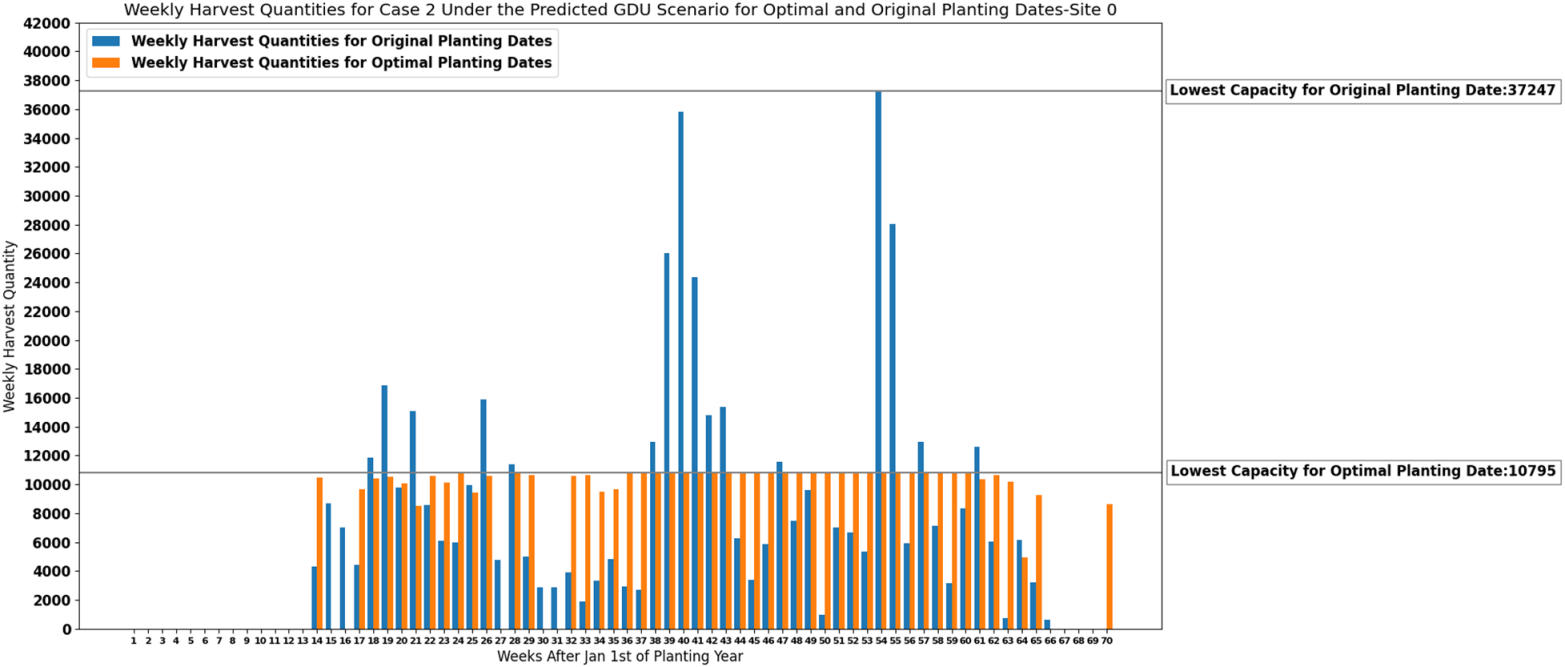
Weekly harvest quantities of site 0 with original capacity of 37,247 and suggested optimal capacity of 10,795 for case 2 under predicted GDU scenario.

**Figure 12:**
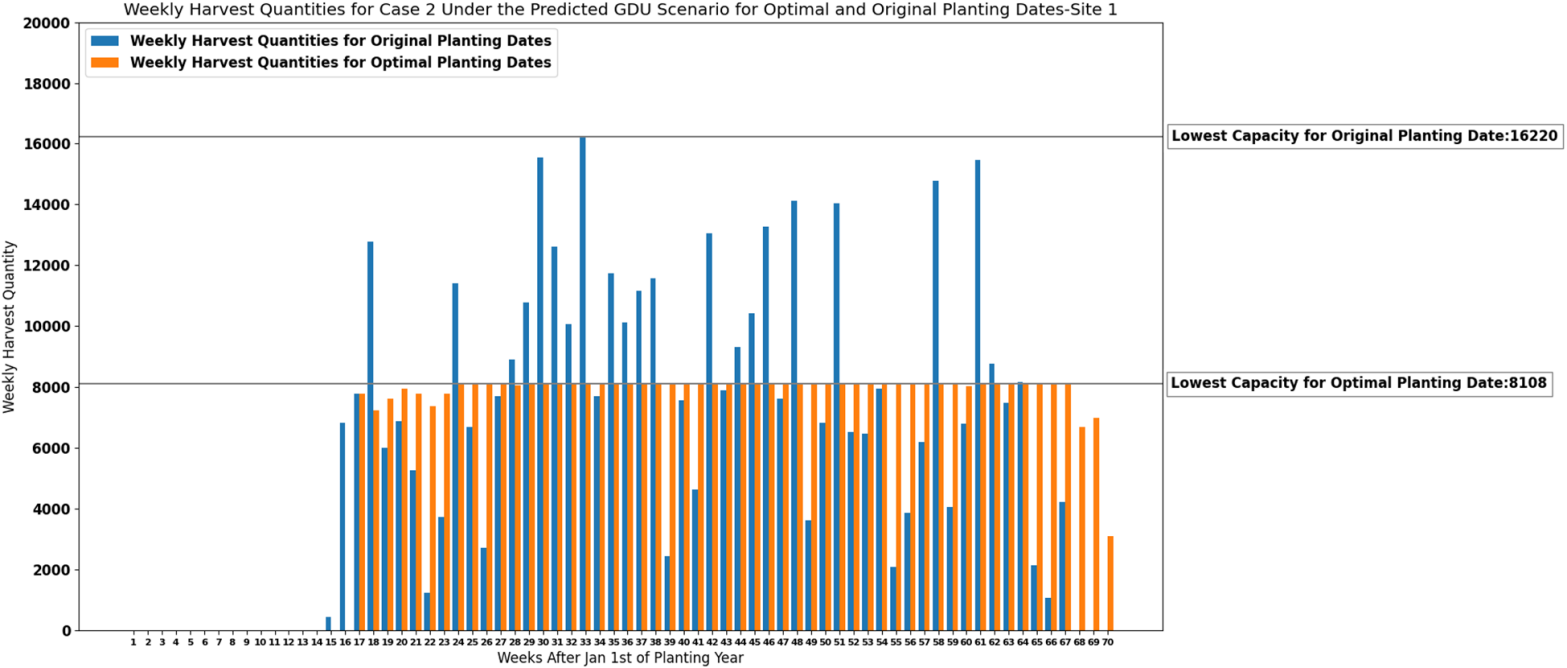
Weekly harvest quantities of site 1 with original capacity of 16,220 and suggested optimal capacity of 8108 for case 2 under predicted GDU scenario.

Figures 11 and 12 demonstrate how our proposed MILP model (30)–(33) for case 2 was able to determine the lowest capacity required for each site under the predicted GDU scenario while successfully scheduling planting and harvesting weeks that resulted in consistent weekly harvest quantities.

**Figure 13:**
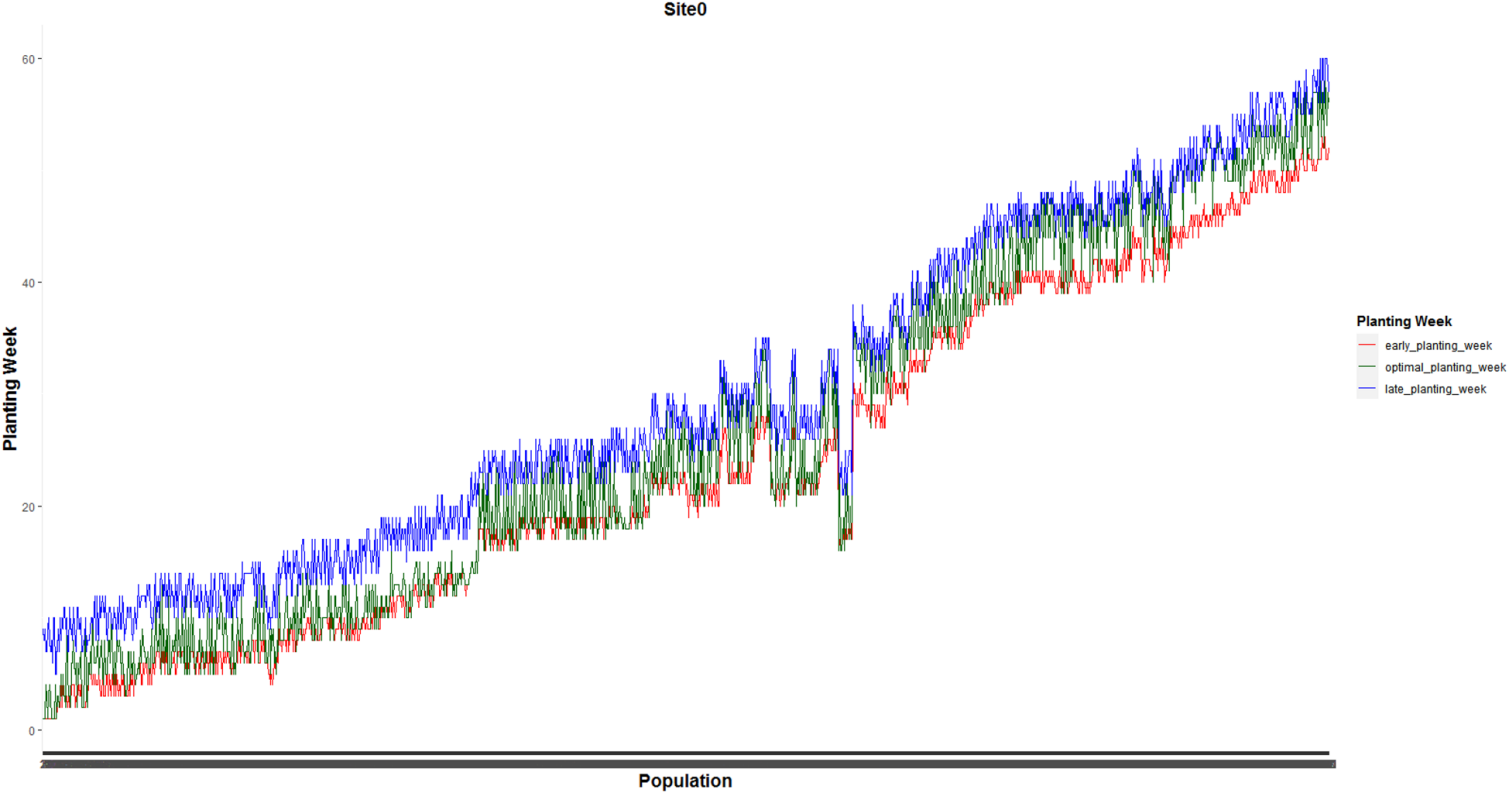
Optimal planting weeks of 1375 seed populations planted in site 0 under the predicted GDU scenario for case 2.

**Figure 14:**
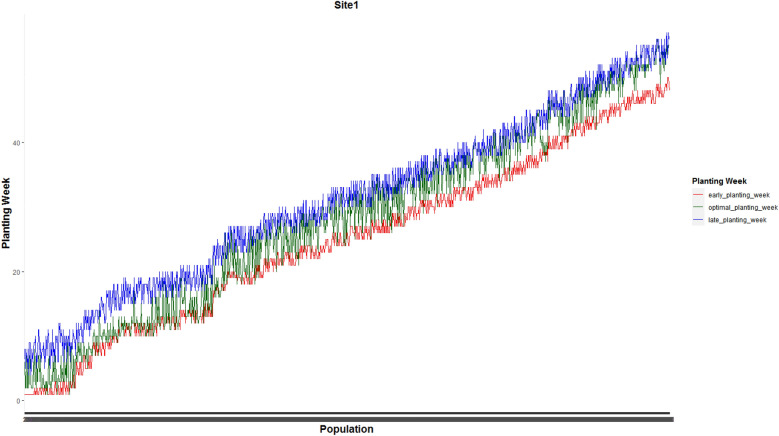
Optimal planting weeks of 1194 seed populations planted in site 1 under the predicted GDU scenario for case 2.

Optimal planting weeks of 1375 seed populations planted in site 0 and 1194 seed populations planted in site 1 for case 2 under the predicted GDU scenario are shown in Figs. 13 and 14 respectively. These figures also show that the optimal planting weeks suggested by our proposed MILP model (30)–(33) are between the early and late planting weeks.

### Results of the stochastic model for case 2

**Figure 15:**
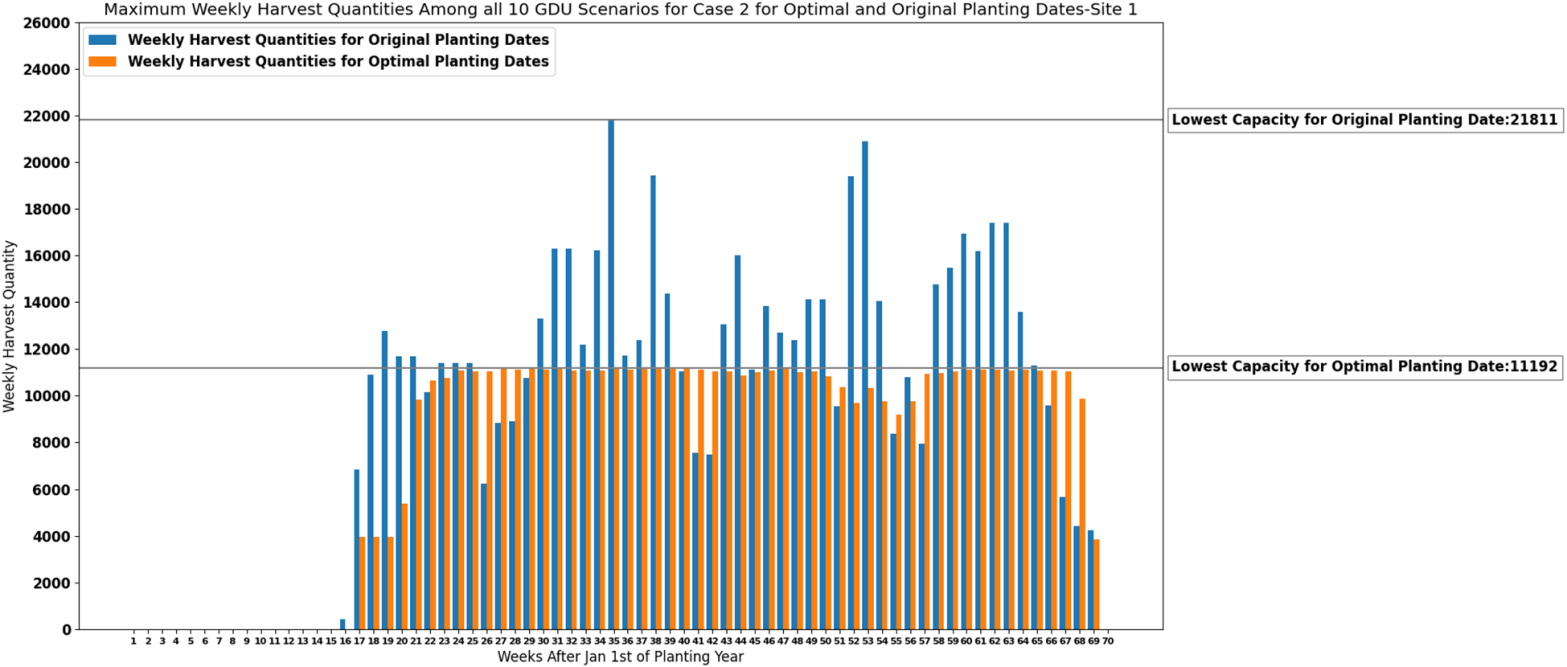
Maximum weekly harvest quantities among all 10 GDU scenarios of site 1 with original capacity of 21,811 and suggested optimal capacity of 11,192 for case 2.

This subsection presents the results of our heuristic algorithm for case 2 where we need to determine the lowest capacity required for each site. As it was discussed in “Results of the stochastic model for case 1” section here we also solved case 2 only for site 1 considering multiple GDU scenarios. Figure 15 shows the maximum weekly harvest quantities among all 10 GDU scenarios for optimal and original planting dates and their lowest capacities required for site 1. In order to show that the results are reasonable we considered weeks 20, 21, and 68 and tried to move the planting dates of the populations with the lowest harvest quantities corresponding to week 20, and week 21 to one week before and the planting date of the population with the lowest harvest quantity corresponding to week 68 to one week after to see how maximum weekly harvest quantities among all 10 GDU scenarios would change and whether these changes would give us better results. The information of those populations with the lowest harvest quantities and how changing their planting dates could affect the results are presented in Table 8 for target weeks of 20, 21, and 68.

**Table 8:** Information of the population IDs 70, 41, and 1063 with the lowest harvest quantities among all populations corresponding to target weeks 20, 21, and 68 and how changing their planting dates to other weeks can affect the results.

Target week	20	21	68
Population ID	70	41	1063
Early planting week	3	1	43
Late planting week	7	6	52
Harvest quantity	70	102	59
Optimal planting week	6	5	52
New planting week	5	4	Not possible
Optimal HQs for weeks of 17/18/19/20/21, and 17/18/19/20/21/22	3958/3958/3958/5391/9851	3958/3958/3958/5391/9851/10657	Not applicable
HQs after change of planting week for weeks of 17/18/19/20/21, and 17/18/19/20/21/22	No change	No change	Not applicable

The lowest storage capacity required for site 1 under multiple GDU scenarios for optimal and original planting dates are presented in the following table (Table 9).

**Table 9:** The lowest capacity required for site 1 under multiple GDU scenarios for optimal planting dates and original planting dates.

Site	Lowest capacity for optimal planting	Lowest capacity for original planting
1	11,192	21,811

**Figure 16:**
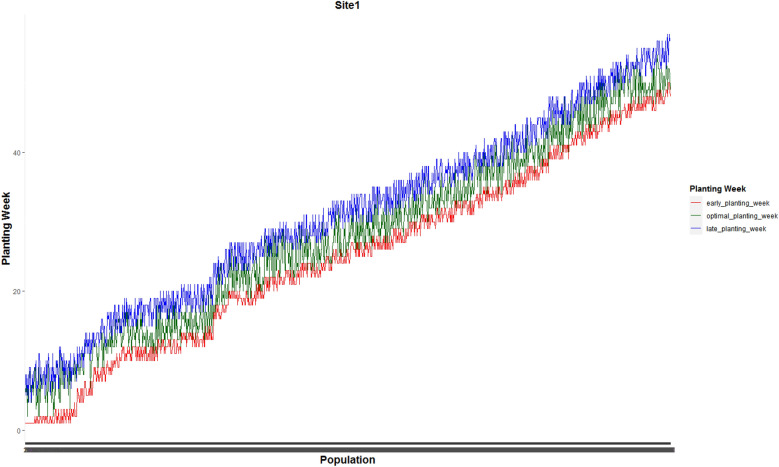
Optimal planting weeks of 1194 seed populations planted in site 1 for all 10 GDU scenarios for case 2.

Our heuristic algorithm resulted in one set of planting dates for all 10 GDU scenarios which is shown in Fig. 16, and ten sets of harvesting weeks which can easily be calculated for each GDU scenario because the populations need to be harvested as soon as they earn their required GDUs.

## Evaluation of the proposed heuristic algorithm

In this section, we evaluate the performance of the proposed heuristic algorithm which was presented in “Heuristic algorithm” section to solve the corn scheduling problem considering multiple GDU scenarios for both storage capacity cases. To this end we solve a small problem for only storage capacity case 1 using both proposed heuristic algorithm (1) and MILP model (19)–(25). The proposed MILP model (19)–(25) with $N \times K \times T + N \times T + K \times T + T$ decision variables which is equal to 920,150 for 1194 populations (N) harvested in 70 weeks (T) under multiple GDU scenarios for 10 years (K) in site 1 can not be solved using the existing branch-and-bound algorithms due to the lack of computational power. So, to evaluate the performance of the proposed heuristic algorithm (1), we created a small problem and instead of using the whole 1194 populations planted in site 1, we only used 100 populations and solved the problem using both heuristic algorithm and MILP model. The MILP model for 100 populations with 77,770 decision variables was run in MATLAB R2018a and solved with MILP commercial solver Gurobi Optimizer. The MILP solver was not able to find a feasible solution and converge even for 100 populations in 72 h. That might be the reason the solution of the MILP model is not satisfactory compared to the solution of the heuristic algorithm proposed specifically to solve the multi GDU scenario.

**Figure 17:**
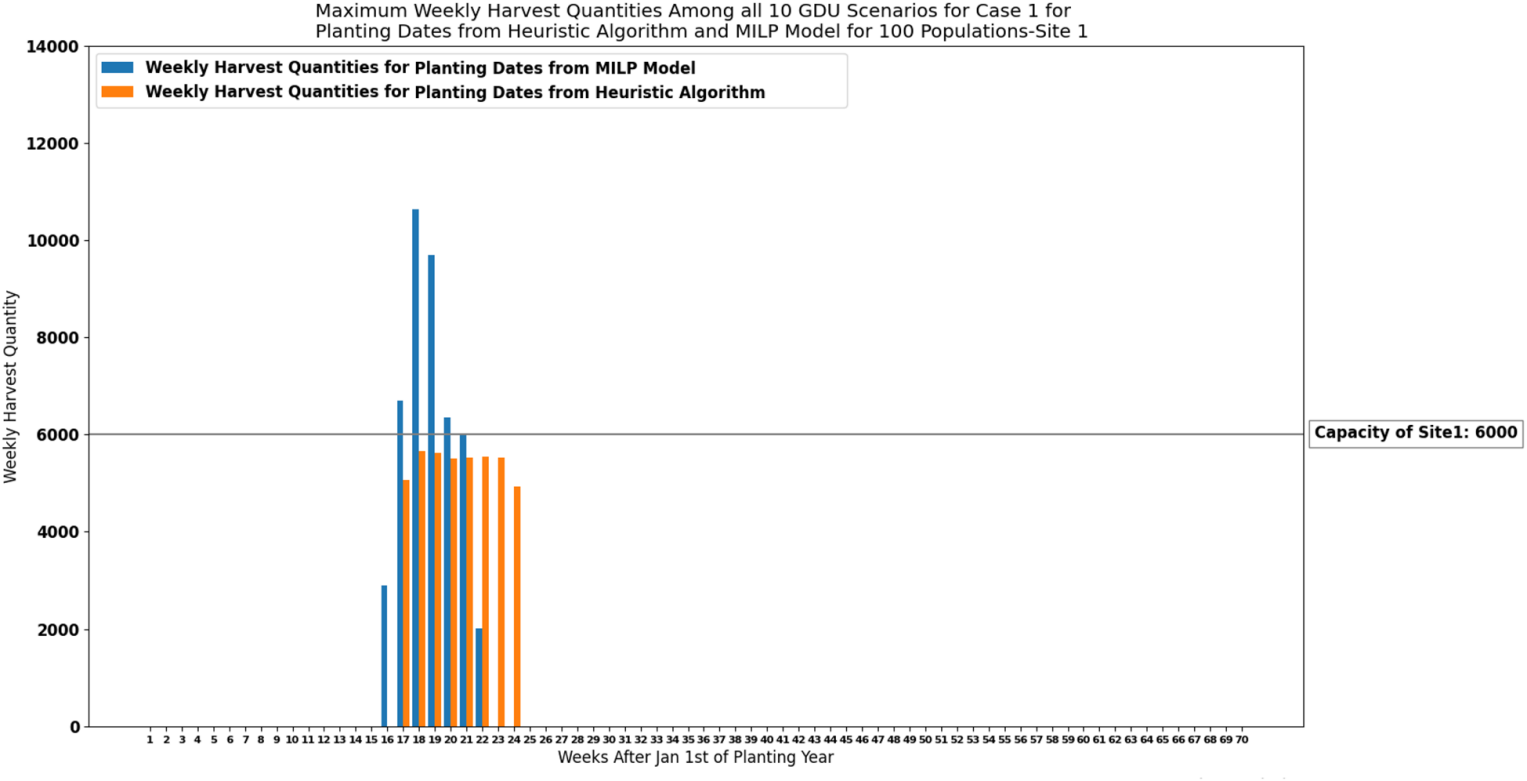
Maximum weekly harvest quantities among all 10 GDU scenarios of site 1 with a capacity of 6000 ears for case 1 for planting dates obtained from the heuristic algorithm and MILP model considering 100 populations. The solution from the MILP model violated the capacity constraint because the MILP solver was unable to find a feasible solution within the 72-hour time limit.

Maximum weekly harvest quantities among all 10 GDU scenarios for planting dates obtained from the heuristic algorithm (1) and MILP model (19)–(25) are shown in Fig. 17 for site 1. Run-times and objective values (sum of absolute differences between maximum weekly harvest quantities among all 10 GDU scenarios and the capacity) of the heuristic algorithm and MILP model using 100 populations from site 1 are presented in Table 10. As it is shown in Fig. 17 and Table 10, the heuristic algorithm has better performance in terms of consistent weekly harvest quantities which are below the capacity and run time.

**Table 10:** Run-times and objective values (sum of absolute differences between maximum weekly harvest quantities among all 10 GDU scenarios and the capacity) of the heuristic algorithm and MILP model using 100 populations from site 1.

Planting date	Run-time (h)	Sum of absolute differences
Heuristic algorithm	0.6	4614
MILP model	72	16,491

## Discussion and conclusion

In this paper, we proposed two MILP models and a heuristics algorithm to help growers and farmers schedule planting and harvesting dates of different corn populations to have consistent harvest quantities that are below the storage capacity of the site for two storage capacity cases considering a deterministic GDU scenario and multiple GDU scenarios together.

Considering a deterministic GDU scenario, the results of the proposed MILP models show that the models can successfully schedule the planting and harvesting dates of diverse seed populations with various planting windows, required GDUs, and harvest quantities planted in two sites in a way that there is no overflow of the capacity for case 1 and a consistent weekly number of ears for both cases. As it was shown in Figs. 5, 6, 11 and 12 the models also could improve the results over the original planting dates provided by the challenge in terms of avoiding overflowing capacity for case 1, suggesting much less lowest capacity required for both sites for case 2, and consistency of weekly harvest quantities for both cases. As it was mentioned before having no carry over the storage capacity can help farmers avoid having to dump harvested crops or leave crops unharvested. The results assure that the proposed MILP models can maximize the benefit by having no carry over of the storage capacities for case 1, and decreasing the lowest capacities required from 37,247 and 16,220 using original planting dates to 10,795 and 8108 using optimal planting dates for site 0 and site 1 respectively for case 2.

Running our MILP models considering a deterministic GDU scenario (predicted GDU) for different amounts of GDUs indicates that the low average temperature or low daily GDUs makes the optimization models infeasible and prevents us from harvesting the whole populations in 70 weeks as the corn populations can not accumulate their required GDUs to reach full maturity. Moreover, the results of our proposed MILP models indicate that different weather conditions or GDU quantities affect the number of harvesting weeks and harvest quantities. These explain why we proposed a heuristic algorithm to solve the problem considering multiple GDU scenarios at the same time. Additionally, the results from the proposed MILP model for case 2 considering a deterministic GDU scenario reveal that the amount of GDU units or weather condition also affects the lowest capacity required and the lower GDU units (lower average temperature) resulted in higher capacity required. It is due to the fact that corn populations accumulate their required GDUs slower, and as a result, higher number of corn populations should be harvested in later weeks. Therefore, higher storage capacity is required due to the limited number of harvesting weeks. As it was described in “Optimization models” section, the proposed MILP models considering multiple GDU scenarios for both cases have $N \times K \times T + N \times T + K \times T + T$ decision variables which is equal to 1,059,520 and 920,150 variables for site 0 and site 1 respectively and they require considerable amount of computational power which makes it infeasible using exact algorithms (*N*, *K*, and *T* are the number of seed populations in each site, number of years of GDU scenarios, and number of harvesting weeks respectively). As such, we proposed a new heuristic algorithm based on simulated annealing to solve the problem. The proposed heuristic algorithm only has *N* decision variables which is equal to the number of seed populations in each site (1375 for site 0 and 1194 for site 1) and took 20 hours to solve each case in python. To evaluate the performance of the proposed heuristic algorithm, we solved a small problem with 100 seed populations for only storage capacity case 1 using both proposed heuristic algorithm (1) and MILP model (19)–(25). The results show that even for a small problem the proposed heuristic algorithm not only has lower computational time but also it has better performance in terms of consistent weekly harvest quantities which are below the capacity.

The different results from cases 1 and 2 raise an interesting question of cost-benefit analysis. On the one hand, case 2 requires storage capacities of 10,795 (site 0) and 8108 (site 1) considering predicted GDU scenario and 11,192 (site 1) considering multiple GDU scenarios, compared with the capacities of 7000 (site 0) and 6000 (site 1) for case 1. On the other hand, the higher capacity in case 2 allows for a total harvest quantity of 923,412 (sites 0 and 1 combined), compared with the total harvest quantity of 657,546 in case 1. Given the cost of a higher storage capacity and the benefit of an increase harvest quantity, we would be able to determine if case 2 is economically more beneficial than case 1.

Since the proposed planting date scheduling models depend on the GDU prediction model performance, we suggest to improve the accuracy of the GDU prediction model with advanced machine learning models such as transformers. Additionally, as it was mentioned in previous sections one of the assumptions of the 2021 Syngenta crop challenge was to harvest the corn hybrids as soon as they reach their required GDUs. For the future work the models can be modified to allow corns to be harvested up to a certain time after they reach maturity rather than being immediately harvested. Moreover, another important criterion which was not required for the challenge and should be taken into account is having consecutive harvesting weeks and the model can be modified to produce the harvesting weeks that are consecutive. We hope that our work leads to the advancement of crop planting and harvest scheduling to benefit plant science as a whole.

## Data Availability

The data analyzed in this study was provided by Syngenta AG company for 2021 Syngenta Crop Challenge. We accessed the data through annual Syngenta Crop Challenge. During the challenge, September 2020 to January 2021, the data was open to the public. Data cannot be shared publicly because of non-disclosure agreement. Data are available from Syngenta (contact via https://www.ideaconnection.com/syngenta-crop-challenge/challenge.php) for researchers who meet the criteria for access to confidential data.
